# Do carboximide–carboxylic acid combinations form co-crystals? The role of hydroxyl substitution on the formation of co-crystals and eutectics

**DOI:** 10.1107/S2052252515002651

**Published:** 2015-04-10

**Authors:** Ramanpreet Kaur, Raj Gautam, Suryanarayan Cherukuvada, Tayur N. Guru Row

**Affiliations:** a Solid State and Structural Chemistry Unit, Indian Institute of Science, Bengaluru 560 012, India

**Keywords:** crystal structure, carboxylic acids, carboximides, co-crystals, eutectics

## Abstract

Carboxylic acids, amides and imides are key organic systems which provide understanding of molecular recognition and binding phenomena important in biological and pharmaceutical settings. In this context, studies of their mutual interactions and compatibility through co-crystallization may pave the way for greater understanding and new applications of their combinations. Extensive co-crystallization studies are available for carboxylic acid/amide combinations, but only a few examples of carboxylic acid/imide co-crystals are currently observed in the literature. The non-formation of co-crystals for carboxylic acid/imide combinations has previously been rationalized, based on steric and computed stability factors. In the light of the growing awareness of eutectic mixtures as an alternative outcome in co-crystallization experiments, the nature of various benzoic acid/cyclic imide combinations is established in this paper. Since an additional functional group can provide sites for new intermolecular inter­actions and, potentially, promote supramolecular growth into a co-crystal, benzoic acids decorated with one or more hydroxyl groups have been systematically screened for co-crystallization with one unsaturated and two saturated cyclic imides. The facile formation of an abundant number of hydroxybenzoic acid/cyclic carboximide co-crystals is reported, including polymorphic and variable stoichiometry co-crystals. In the cases where co-crystals did not form, the combinations are shown invariably to result in eutectics. The presence or absence and geometric disposition of hydroxyl functionality on benzoic acid is thus found to drive the formation of co-crystals or eutectics for the studied carboxylic acid/imide combinations.

## Abstract 2

Cocrystallization of cyclic imides with benzoic acid and some of its hydroxyl analogues resulted in cocrystals and eutectics in a mutually exclusive manner depending on the presence or absence and position of the hydroxyl group and consequent supramolecular effect for different combinations.

## 1. Introduction   

There is a renewed interest in understanding the chemical factors that govern the phenomenon of co-crystallization (Cherukuvada & Row, 2014 ▶; Prasad *et al.*, 2014 ▶; Wood *et al.*, 2014 ▶; Mukherjee *et al.*, 2014 ▶; Aitipamula, Chow & Tan, 2014 ▶; Bučar *et al.*, 2013 ▶; Seaton & Parkin, 2011 ▶; Braga *et al.*, 2010 ▶; Friščić & Jones, 2009 ▶; Lu *et al.*, 2008 ▶; Aakeröy *et al.*, 2008 ▶; Chadwick *et al.*, 2007 ▶; Friščić *et al.*, 2006 ▶; Shan *et al.*, 2002 ▶), owing largely to its potential importance in the pharma­ceutical industry (Cherukuvada & Nangia, 2014 ▶; Aakeröy *et al.*, 2014 ▶; Brittain, 2012 ▶; Babu & Nangia, 2011 ▶; Chen *et al.*, 2011 ▶; Schultheiss & Newman, 2009 ▶; Shan & Zaworotko, 2008 ▶; Blagden *et al.*, 2007 ▶; Trask & Jones, 2005 ▶). Co-crystallization is a supramolecular reaction to form multi-component organic adducts such as co-crystals, solid solutions, eutectics *etc.* (Cherukuvada & Nangia, 2014 ▶; Cherukuvada & Row, 2014 ▶; Prasad *et al.*, 2014 ▶). Whether a co-crystal or a eutectic is formed depends on the dominance of hetero- and homomolecular interactions, respectively, for a given combination of materials. Several aspects play a role in the formation of co-crystals and eutectics, such as the nature and influence of the molecular components in invoking intermolecular interactions and supramolecular synthons, functional group disposition and complementarity, interaction strength, and efficient packing. However, there is no general recipe to obtain selectively or reliably either co-crystals or eutectics on demand. Investigations into this effect are important to save time, money and effort in targeted co-crystal or eutectic screens.

The literature describes numerous failed co-crystallization experiments (for example, Alhalaweh *et al.*, 2012 ▶; Arenas-García *et al.*, 2012 ▶; Seaton & Parkin, 2011 ▶; Caira *et al.*, 2012 ▶; Mohammad *et al.*, 2011 ▶; Karki *et al.*, 2010 ▶), which did not investigate the potential formation of eutectics. Given their potential importance in the pharmaceutical and materials fields (Cherukuvada & Nangia, 2014 ▶; Griffini *et al.*, 2014 ▶; Huang *et al.*, 2013 ▶; Yan *et al.*, 2011 ▶; Morimoto & Irie, 2010 ▶; Karaipekli & Sarı, 2010 ▶; Schultheiss & Newman, 2009 ▶; Moore & Wildfong, 2009 ▶), there is a need for more studies of the attributes that govern co-crystal/eutectic formation. Exploring systems with subtle differences in hydrogen-bonding functional groups can serve as a lead, since these groups can steer supramolecular growth as either a co-crystal or a eutectic for a given combination (Cherukuvada & Nangia, 2014 ▶; Cherukuvada & Row, 2014 ▶; Prasad *et al.*, 2014 ▶). In this context, we have selected cyclic carboximides for an in-depth co-crystallization study with carboxylic acids. The latter class of compounds has a wide variety of applications, particularly in the pharmaceutical field, as drugs, salts and co-formers, excipients *etc.* (Cherukuvada & Nangia, 2014 ▶; Aitipamula, Wong *et al.*, 2014 ▶; Ballatore *et al.*, 2013 ▶; Losev *et al.*, 2013 ▶; Ebenezer & Muthiah, 2012 ▶, Reddy *et al.*, 2011 ▶; Seaton, 2011 ▶; Moffat *et al.*, 2011 ▶; Rowe *et al.*, 2006 ▶; Caira *et al.*, 1995 ▶; Gould, 1986 ▶). Likewise, amide (primary and secondary) and imide functionalities are found in several drugs and are amenable to both salt and co-crystal formation (Buist *et al.*, 2013 ▶; Sanphui *et al.*, 2013 ▶; Nanubolu *et al.*, 2012 ▶; Cherukuvada & Nangia, 2012 ▶; Moffat *et al.*, 2011 ▶; Cherukuvada *et al.*, 2011 ▶). Therefore, the study of the interactions and compatibility of amide/imide–carboxylic acid combinations has direct practical significance.

**Figure 1:**
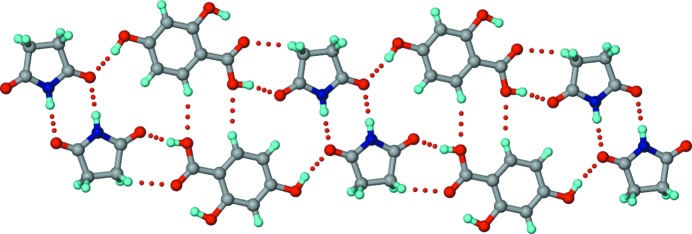
The succinimide–2,4-dihydroxybenzoic acid (SM–24DHBA) co-crystal (Moragues-Bartolome *et al.*, 2012 ▶). The structure shows acid-flanked imide homodimers (*T*_homo_ units, Fig. 2 ▶) propagated by hydroxyl–carbonyl hydrogen bonds (dotted lines) involving the *para*-hydroxyl group of 24DHBA. We designate this as polymorph I, with polymorph II reported herein.

**Figure 2:**
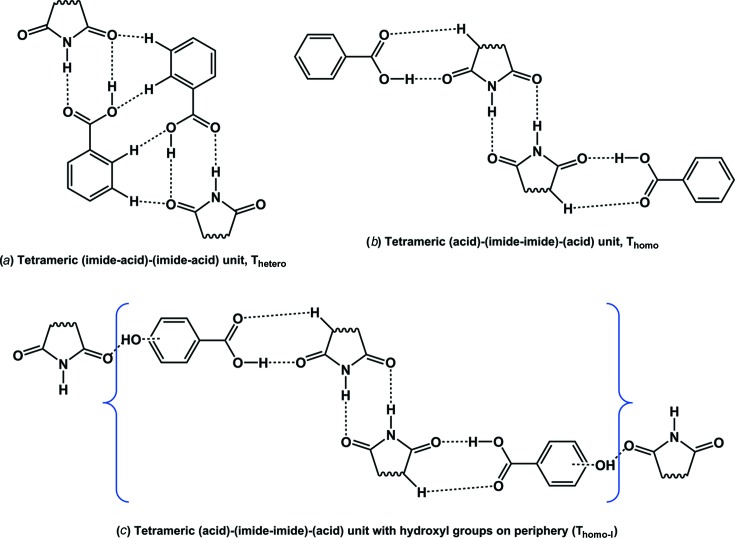
(*a*) The tetrameric unit of two carboxylic acid–imide heterodimers. (*b*) The tetrameric unit of an acid-flanked imide homodimer. Both (*a*) and (*b*) have been calculated to be less stable (Moragues-Bartolome *et al.*, 2012 ▶) and hence less likely to occur in co-crystals. (*c*) The reported SM–24DHBA co-crystal shows the *T*_homo_ unit, with the crucial stabilization and propagation of the unit *via para*-OH⋯carbonyl (imide) hydrogen bonds. Dashed lines indicate hydrogen bonds.

Co-crystallization of carboxylic acids with amides has been studied extensively (Cherukuvada & Row, 2014 ▶; Moragues-Bartolome *et al.*, 2012 ▶; Kaur & Row, 2012 ▶; Babu *et al.*, 2012 ▶; Cherukuvada & Nangia, 2012 ▶; Reddy *et al.*, 2007 ▶; McMahon *et al.*, 2005 ▶; Leiserowitz & Nader, 1977 ▶), whereas only limited studies of carboxylic acid/imide combinations are found in the literature. The prospect for co-crystal formation involving carboximide and carboxylic acid groups has been considered (Moragues-Bartolome *et al.*, 2012 ▶), and it was suggested that these groups are not expected to interact within co-crystals. Moragues-Bartolome *et al.* (2012 ▶) reported the co-crystallization of saturated cyclic imides (succinimide and glutarimide) with a variety of aliphatic and aromatic monocarboxylic acids and obtained only one co-crystal, namely succinimide–2,4-di­hydroxy­benzoic acid (SM–24DHBA), as shown in Fig. 1 ▶. Based on the steric hindrance of the extra imide carbonyl group and the low stabilizing features of imide–acid and acid-supported imide–imide hydrogen-bonding motifs (named *T*_hetero_ and *T*_homo_ units, respectively; Fig. 2 ▶) compared with amides, they deduced that the formation of cyclic imide–carboxylic acid co-crystals is unlikely. The study considered carboxylic acid–imide combinations, of which the majority had hydrogen-bond acceptor groups (fluoro, nitro *etc.*) on the acid partner. Since the hydrogen-bond demands of the extra imide carbonyl acceptor cannot be complemented by acceptor groups on the partner molecules, co-crystal formation is curtailed due to high-energy interactions (repulsions) associated with acceptor–acceptor (carbonyl *versus* fluoro/nitro) combinations. It is understandable that a hydrogen-bond donor like hydroxyl can satisfy the imide carbonyl and therefore lead to the SM–24DHBA co-crystal (Figs. 1 ▶ and 2 ▶ *c*).

**Figure 3:**
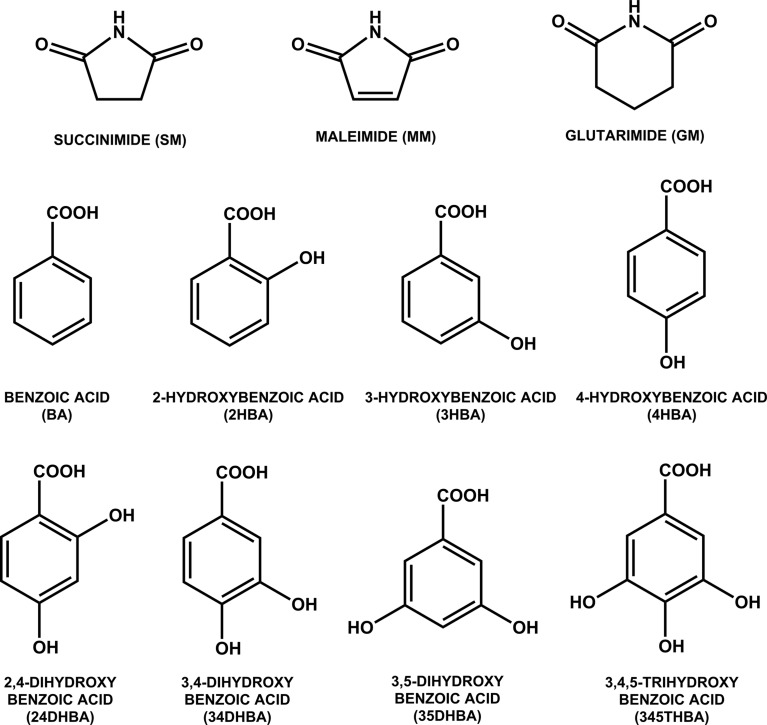
Molecular structures and acronyms. Moragues-Bartolome *et al.* (2012 ▶) previously obtained a co-crystal for the SM–24DHBA combination and reported that the SM–BA and GM–BA combinations lead to physical mixtures.

**Figure 4:**
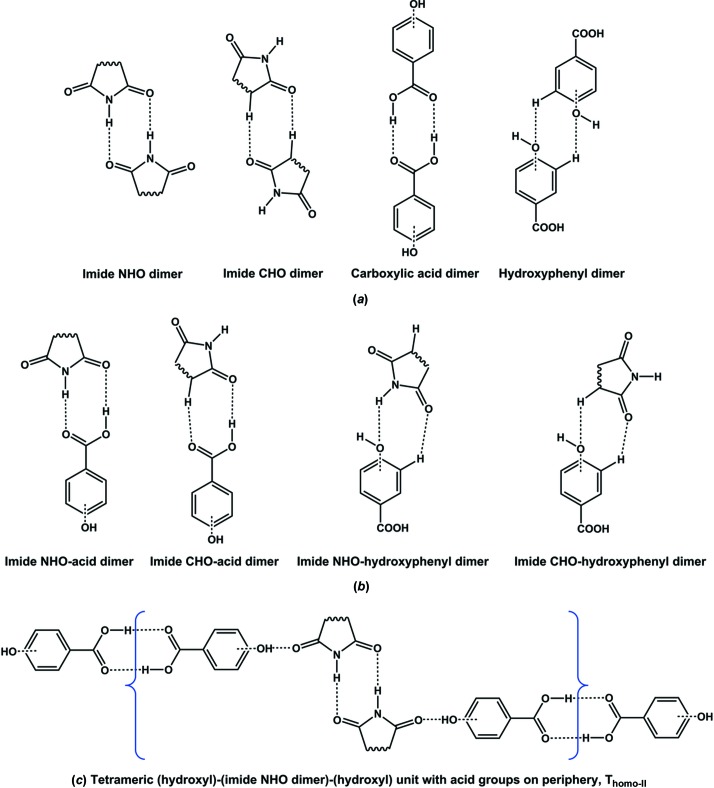

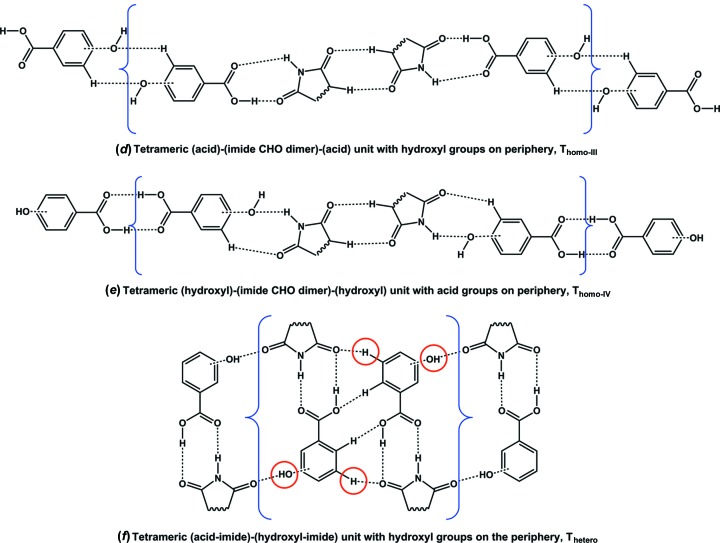
(*a*) The homodimeric and (*b*) the heterodimeric primary recognition units of cyclic imide–hydroxybenzoic acid combinations. (*c*) A tetrameric unit comprising a hydroxyl-supported imide homodimer (*T*_homo_-II) can propagate through carboxylic acid homodimers to form co-crystals. (*d*) and (*e*) Similarly, the progression of tetrameric units can result in co-crystals. (*f*) Propagation of the *T*_hetero_ unit can take place through OH substitution at *meta*-positions (indicated by red circles), which confers stronger O—H_hydroxyl_⋯O=C_imide_ auxiliary interactions (compared with C—H⋯O=C) and therefore gives rise to co-crystals. Dashed lines indicate hydrogen bonds. Eutectics, which are hallmarked by finite and discrete units, can be formed for combinations where tetrameric units are not stabilized and/or propagated.

In the context of eutectics as alternative supramolecular assemblies to co-crystals (Cherukuvada & Nangia, 2014 ▶; Cherukuvada & Row, 2014 ▶; Prasad *et al.*, 2014 ▶), and with the hypothesis that auxiliary interactions play a crucial role, we undertook the task of establishing the nature of different imide–carboxylic acid combinations. We selected for study three cyclic imides (succinimide, glutarimide and maleimide, which is unsaturated) and seven hydroxybenzoic acids, in addition to the parent benzoic acid (Fig. 3 ▶). The rationale for the selection of hydroxybenzoic acids is that the presence of hydroxyl group(s) on the benzoic acid molecule would instigate auxiliary interactions with the extra imide carbonyl, thereby facilitating supramolecular growth units beyond *T*_hetero_ or *T*_homo_ units (Fig. 2 ▶). We devised a scheme of dimeric and tetrameric hydrogen-bonded units that could form in carboxylic acid/imide combinations (Fig. 4 ▶). We perceive that the supramolecular propagation of these units should lead to the formation of co-crystals, with eutectics being formed otherwise (Fig. 4 ▶). We were successful in obtaining several co-crystals and eutectics of cyclic imide–hydroxybenzoic acids. We also obtained a new polymorph for the reported succin­imide–2,4-dihydroxybenzoic acid co-crystal (SM–24DHBA) and a new dimorphic pair of 2:1 succinimide–3,4,5-tri­hydroxy­benzoic acid co-crystals. This work demonstrates that the presence or absence of hydroxyl group(s) dictates the formation or non-formation of imide–carboxylic acid co-crystals in the systems studied here.

## 2. Results and discussion   

We performed co-crystallization by solution crystallization, following both neat (Trask & Jones, 2005 ▶) and liquid-assisted grinding (Friščić *et al.*, 2006 ▶; Shan *et al.*, 2002 ▶) of all combinations (see §S1 in the supporting information for experimental details). Ground products were subjected to powder X-ray diffraction (PXRD) and melting-point determination to ascertain co-crystal/eutectic formation, on the basis that the former exhibit distinct PXRD patterns and melting behaviour while the latter display only a depression of the melting point compared with the parent materials (Cherukuvada & Nangia, 2014 ▶; Cherukuvada & Row, 2014 ▶; Prasad *et al.*, 2014 ▶). X-ray single-crystal structures were determined for co-crystals (except for a few where suitable single crystals were not obtained) and phase diagrams were constructed for eutectics. The results of the co-crystallization experiments are listed in Table 1 ▶. Benzoic acid and the mono-hydroxybenzoic acids, except the 4-hydroxy isomer, gave eutectics with all three cyclic imides (Table 1 ▶). Along with 4-hydroxybenzoic acid (4HBA), all the di- and tri-hydroxybenzoic acids resulted in co-crystals with all three imides. A new polymorph of the reported succinimide–24DHBA co-crystal and a dimorphic pair of 2:1 succinimide–345THBA co-crystals were also obtained (Table 1 ▶). Crystallographic parameters of the co-crystals are given in §S2 of the supporting information. Comparison of the experimental PXRD patterns with the respective parent materials is provided in §§S3 and S4 of the supporting information in order to differentiate the co-crystal- and eutectic-forming combinations.

### 2.1. Rationale for the formation of co-crystals or eutectics   

The primary supramolecular recognition units in an imide–carboxylic acid combination are imide–imide, acid–acid and acid (COOH)–imide (CONH or COCH) centrosymmetric ring dimer motifs (Figs. 4 ▶ *a* and 4 ▶ *b*). If these units, either homo- or heterodimers, can extend through auxiliary interactions (such as O—H/C—H_carboxylic acid_⋯O=C_imide_) to form *T*_homo_ or *T*_hetero_ tetramers and then propagate, the formation of a co-crystal is facile, as per Fig. 4 ▶. On the other hand, a eutectic mixture results if the units remain finite and discrete in the supramolecular lattice (Cherukuvada & Nangia, 2014 ▶; Cherukuvada & Row, 2014 ▶; Prasad *et al.*, 2014 ▶). We observed several intriguing results from the co-crystallization experiments: (i) all cyclic imides formed co-crystals with *para*-hydroxy substituted and di- or tri-hydroxy benzoic acids; (ii) non-formation of co-crystals in the case of benzoic acid and *ortho*- or *meta*-hydroxybenzoic acids, which instead formed eutectics; (iii) polymorphism in co-crystals; (iv) variable stoichiometry co-crystals; and (v) diverse co-crystal architectures. These features can be rationalized as follows.

First, the geometric positioning of a *para*-hydroxyl group aptly fits and promotes the supramolecular geometry of the *T*_homo_ unit (Figs. 2 ▶ and 4 ▶) to give co-crystals. By contrast, *ortho*- or *meta*-hydroxyl substitution provides no energetic stabilization to either *T*_hetero_ or *T*_homo_ supramolecular growth units and hence results in eutectic phases with all three cyclic imides. In the *ortho*-position, the hydroxyl group always participates in intramolecular hydrogen bonding with the carboxylic acid (O—H_hydroxyl_⋯O=C_acid_), such that it is unavailable for auxiliary interactions with the imide carbonyl group, and therefore propagation of *T*_hetero_ or *T*_homo_ units does not take place. Although it would seem that the *meta*-hydroxyl substituent could promote the *T*_homo_ unit, geometric reasons appear to resist supramolecular growth into a co-crystal. On the other hand, a *meta*-hydroxyl group fits the geometry and can stabilize the *T*_hetero_ unit. However, the stabilizing interactions from a lone *meta*-hydroxyl group seem to be insufficient and an additional substitution at the other *meta*-position is required for the *T*_hetero_ unit to propagate into a co-crystal (Fig. 4 ▶ *f*). Thus, 35DHBA can distinctly form a *T*_hetero_ unit in its co-crystals and indeed it is found in the 1:1 GM–35DHBA co-crystal (as described later). It should be noted that a *para*-hydroxyl group does not suit the *T*_hetero_ unit and so cannot result in co-crystals for the same geometric reasons. In view of the above, it is obvious that unsubstituted benzoic acid forms only eutectics with the three cyclic imides. The hydrogen-bond demands of the additional strong imide carbonyl may not be satisfied by weak C—H donors (of benzoic acid) nor even by a strong hydroxyl group donor in a certain geometry (*ortho*- or *meta*-position of substituted benzoic acid), such that these combinations cannot make co-crystal growth units and therefore form eutectics.

Secondly, the crystal structure of the reported SM–24DHBA co-crystal (Moragues-Bartolome *et al.*, 2012 ▶) (Fig. 1 ▶) supports our explanation of co-crystal/eutectic formation for different imide–carboxylic acid combinations in this study. Based on the above, it is reasonable to expect that all three imides can form co-crystals with the *para*-hydroxy substituted benzoic acids considered (Table 1 ▶). On the other hand, several co-crystals were obtained with supramolecular patterns different from those illustrated in Figs. 2 ▶ and 4 ▶, and they crystallized in different polymorphs and multiple stoichiometries. The crystal structures of the obtained cyclic imide–hydroxybenzoic acid co-crystals are discussed next, followed by phase diagrams for the eutectic-forming combinations.

### 2.2. Succinimide–hydroxybenzoic acid co-crystals   

#### 2.2.1. 1:1 SM–4HBA   

**Figure 5:**
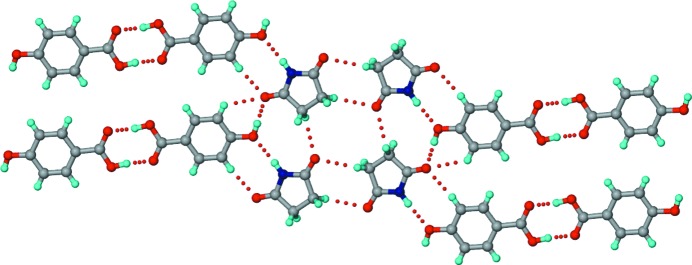
The structure of the 1:1 SM–4HBA co-crystal. *T*_homo_-IV units are connected by 4HBA carboxylic acid homodimers to make parallel tapes that extend into a sheet through O—H_hydroxy_⋯O=C_imide_ and multiple C—H⋯O interactions. The strong imide carbonyl acceptors are involved in multifurcated hydrogen bonds. Dotted lines indicate hydrogen bonds.

In this crystal structure, *T*_homo_-IV units (composed of succinimide C—H⋯O homodimers and imide–hydroxyphenyl heterodimers) propagate into tapes through carboxylic acid dimers (Fig. 5 ▶). Such tapes extend into two-dimensional sheets through O—H_hydroxy_⋯O=C_imide_ and multiple C—H⋯O hydrogen bonds. The hydroxyl group clearly plays a major role in invoking auxiliary interactions and sustaining both the one- and two-dimensional motifs.

#### 2.2.2. 1:1 SM–24DHBA, polymorph II   

**Figure 6:**
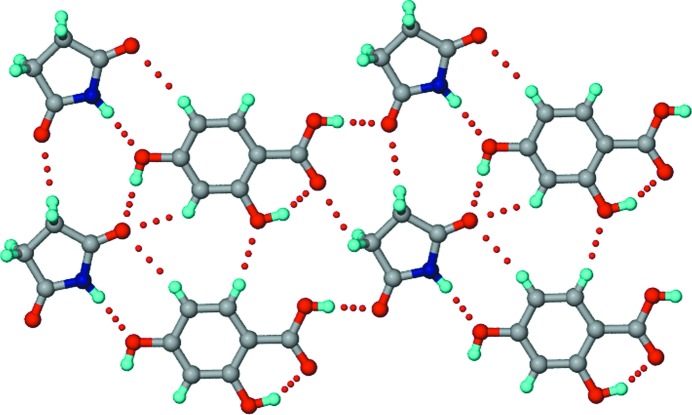
The structure of polymorph II of the 1:1 SM–24DHBA co-crystal. Parallel zigzag tapes formed by imide–hydroxyphenyl heterodimers between SM and 24DHBA molecules extend into a sheet through acid–carbonyl, hydroxyl–carbonyl and C—H⋯O interactions. The *para*-hydroxyl conformation in 24DHBA is *cis* with respect to the carbonyl of the acid group. Dotted lines indicate hydrogen bonds.

Crystallization of a 1:1 SM–24DHBA ground mixture in an effort to reproduce the reported 1:1 co-crystal (polymorph I; Moragues-Bartolome *et al.*, 2012 ▶) resulted in a new polymorph of the co-crystal (polymorph II). This dimorphic pair represents a case of conformational and synthon polymorphism (Aitipamula, Chow & Tan, 2014 ▶; Aitipamula, Wong *et al.*, 2014 ▶). The polymorphs differ in the conformation of the *para*-hydroxyl group, which is *trans* to the carbonyl of the acid group in polymorph I, and *cis* in polymorph II (Figs. 1 ▶ and 6 ▶). Whereas polymorph I shows the acid-flanked imide homodimer (*T*_homo_-I unit, Fig. 1 ▶), polymorph II displays no imide or acid homodimer (Fig. 6 ▶). Instead, imide–hydroxyphenyl SM–24DHBA heterodimers permit the extra imide carbonyl and free acid groups to form hydrogen bonds with each other, propagating into a zigzag tape. Such tapes extend into a sheet structure through hydroxy–carbonyl_imide_ and C—H⋯O interactions. The absence of strong imide N—H⋯O or acid homodimers or imide–acid heterodimers in the co-crystal seems to be compensated by maximal intermolecular hydrogen bonding.

#### 2.2.3. 1:2 SM–34DHBA   

**Figure 7:**
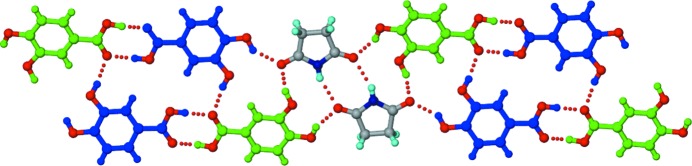
The structure of the 1:2 SM–34DHBA co-crystal. *T*_homo_-II units propagate into tapes through acid homodimers formed by symmetry-independent 34DHBA molecules (shown in different colours), which have different hydroxyl conformations. Dotted lines indicate hydrogen bonds.

Crystallization of a 1:1 SM–34DHBA ground mixture from acetonitrile resulted in a co-crystal with 1:2 stoichiometry. In the crystal structure of 1:2 SM–34DHBA, N—H⋯O dimers between inversion-related SM molecules make *T*_homo_-II units with their peripheral carbonyls hydrogen-bonded to hydroxyl groups of 34DHBA molecules (Fig. 7 ▶). These units propagate through acid homodimers between symmetry-independent 34DHBA molecules which have differences in their hydroxyl conformations (*cis–cis* in one case and *trans–trans* in the other with respect to the carbonyl of the acid group).

#### 2.2.4. 1:3:3 SM–35DHBA–H_2_O   

**Figure 8:**
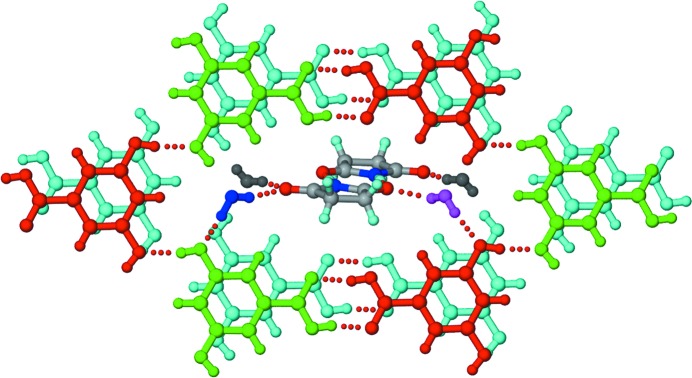
The structure of the 1:3:3 SM–35DHBA–H_2_O co-crystal hydrate. Parallel hexameric motifs formed by symmetry-independent 35DHBA molecules (shown in different colours) make voids for the succinimide and water molecules. Dotted lines indicate hydrogen bonds. The disorder of the H atoms is not shown.

Co-crystallization of SM and 35DHBA was expected to provide a 1:1 co-crystal having exclusively *T*_hetero_ units, as per the geometric features outlined in Fig. 4 ▶. Interestingly, however, a hydrated co-crystal with stoichiometry 1:3:3 SM–35DHBA–H_2_O was obtained upon crystallization from methanol. In the crystal structure, planar hexameric motifs of 35DHBA molecules make voids that are filled by succinimide N—H⋯O dimers and water molecules (Fig. 8 ▶). The co-crystal is stabilized by forming a network of O—H⋯O interactions involving the hydroxyl groups and water molecules. On the basis of constructing an extended in-plane hydrogen-bond network, the hydroxyl groups of one of the three symmetry-independent 35DHBA molecules appear to be disordered.

#### 2.2.5. 2:1 SM–345THBA polymorphs   

**Figure 9:**
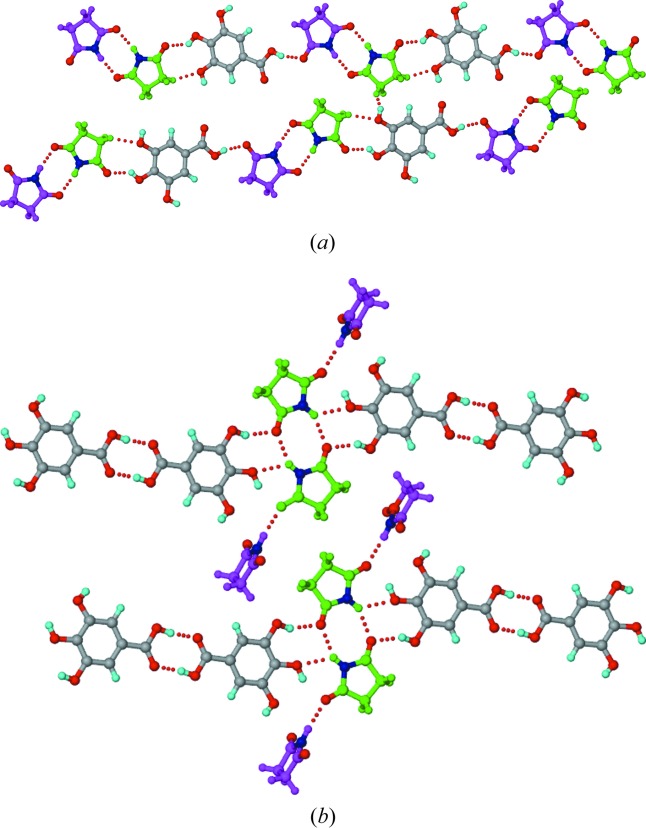
(*a*) Polymorph I of the 2:1 SM–345THBA co-crystal. An infinite tape is formed by N—H⋯O dimers between symmetry-independent SM molecules (shown in different colours), with peripheral imide carbonyls involved in hydrogen bonding with the acid and hydroxyl OH groups of the 345THBA molecules. The hydroxyl groups of 345THBA adopt a *cis–cis–trans* conformation. (*b*) In polymorph II, one of the symmetry-independent SM molecules (shown in green) forms *T*_homo_-II units, and makes an N—H⋯O bond with the other symmetry-independent SM molecule (shown in magenta) through its peripheral imide in an orthogonal fashion. The hydroxyl groups adopt an all *trans* conformation. Dotted lines indicate hydrogen bonds.

Crystallization of a 1:1 SM–345THBA ground mixture from methanol resulted in two polymorphs of a 2:1 co-crystal, designated polymorph I (space group *P*2_1_2_1_2_1_) and polymorph II (space group 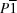). In polymorph I, the hydroxyl groups of 345THBA have a *cis–cis–trans* geometry, while they have an all-*trans* geometry in polymorph II (Fig. 9 ▶). In polymorph I, N—H⋯O dimers between SM molecules permit the peripheral imide carbonyls to accept hydrogen bonds from acid and hydroxyl OH groups and propagate a tape (Fig. 9 ▶ *a*). In polymorph II, N—H⋯O dimers between inversion-related SM molecules make *T*_homo_-II units with the peripheral carbonyls, supported by hydrogen bonds from the hydroxyl groups of 345THBA molecules (Fig. 9 ▶ *b*). Additionally, the outlying imide carbonyl of each SM molecule accepts a hydrogen bond from the imide NH of another SM molecule in an orthogonal manner. This dimorphic pair of co-crystals also exhibits conformational and synthon polymorphism (Aitipamula, Chow & Tan, 2014 ▶; Aitipamula, Wong *et al.*, 2014 ▶).

### 2.3. Maleimide–hydroxybenzoic acid co-crystals   

#### 2.3.1. 1:1 MM–4HBA   

**Figure 10:**
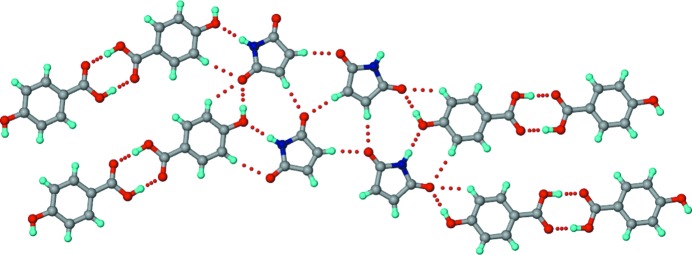
The structure of the 1:1 MM–4HBA co-crystal. Parallel tapes consisting of imide C—H⋯O-connected maleimide molecules and acid homodimers joined by imide–hydroxyphenyl heterodimers extend into a sheet through O—H_hydroxy_⋯O=C_imide_ and multiple C—H⋯O interactions. Dotted lines indicate hydrogen bonds.

Crystallization of a 1:1 MM–4HBA ground mixture from methanol resulted in a 1:1 co-crystal. The structure exhibits similarity to the 1:1 SM–4HBA co-crystal in that the tapes formed by C—H⋯O-connected maleimide molecules and 4HBA carboxylic acid dimers, joined by imide–hydroxyphenyl interactions, extend into a sheet structure through O— H_hydroxyl_⋯O=C_imide_ and multiple C—H⋯O interactions (Fig. 10 ▶). Further, akin to SM–4HBA, the MM–4HBA co-crystal features maximal intermolecular hydrogen bonding to compensate for the lack of strong imide N—H⋯O homodimers.

#### 2.3.2. 1:1 MM–24DHBA   

**Figure 11:**
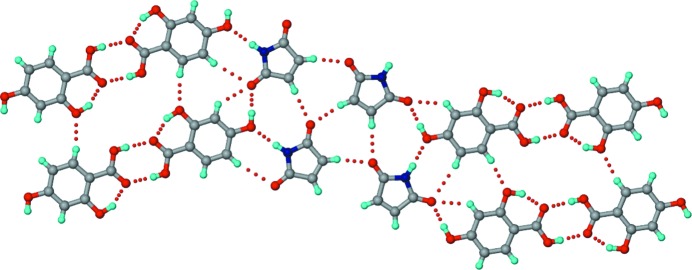
The structure of the 1:1 MM–24DHBA co-crystal. Parallel tapes consisting of imide C—H⋯O-connected maleimide molecules and acid homodimers attached through imide–hydroxyphenyl heterodimers extend into a sheet through O—H_hydroxy_⋯O=C_imide_ and multiple C—H⋯O interactions. Dotted lines indicate hydrogen bonds.

Crystallization of a 1:1 MM–24DHBA ground mixture from methanol resulted in a 1:1 co-crystal. Interestingly, the structure has no resemblance to either of the two 1:1 SM–24DHBA co-crystal polymorphs. Instead, it exhibits similarity with the MM–4HBA and SM–4HBA co-crystals, more so with the former in that both of them lack the centrosymmetric imide C—H⋯O homodimers which are characteristic of the SM–4HBA co-crystal. Overall, the co-crystal forms a sheet structure akin to MM–4HBA, sustained by imide–hydroxyphenyl and C—H⋯O interactions (Fig. 11 ▶).

#### 2.3.3. 1:3:3 MM–35DHBA–H_2_O   

**Figure 12:**
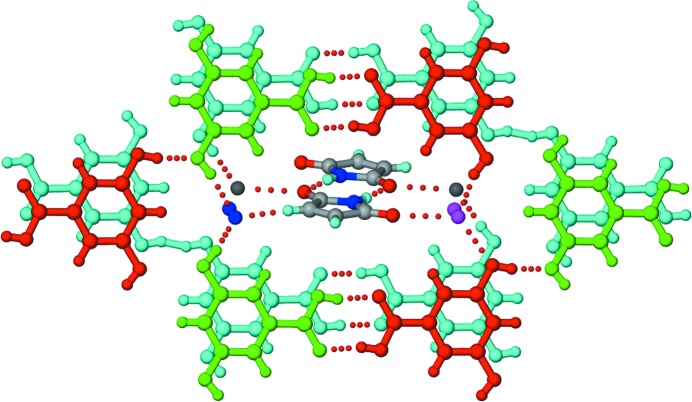
The structure of the 1:3:3 MM–35DHBA–H_2_O co-crystal hydrate. The structure is comparable with that of SM–35DHBA–H_2_O. Parallel hexameric motifs formed by two sets of unique 35DHBA molecules (shown in different colours) make voids for the maleimide and water molecules. Dotted lines indicate hydrogen bonds. The disorder of the H atoms is not shown.

Similar to SM–35DHBA, a co-crystal trihydrate with stoichiometry 1:3:3 MM–35DHBA–H_2_O was obtained when a 1:1 MM–35DHBA ground mixture was crystallized from methanol. The structure is closely comparable with (but not entirely identical to) SM–35DHBA–H_2_O, in which hexameric motifs of 35DHBA molecules make voids for succinimide N—H⋯O dimers and water molecules (Fig. 12 ▶). In this case, all of the hydroxyl groups and water molecules appear to be disordered within the planar hydrogen-bond networks. Compared with SM–35DHBA, the layers of 35DHBA molecules are aligned slightly differently, as a result of accommodating the MM molecule rather than the SM molecule within the voids.

#### 2.3.4. MM–34DHBA and MM–345THBA combinations   

Although no crystal structures could be determined because of a lack of diffraction-quality single crystals, distinct PXRD patterns compared with their parent compounds establish these as co-crystal-forming combinations (see supporting information).

### 2.4. Glutarimide–hydroxybenzoic acid co-crystals   

#### 2.4.1. 1:2 GM–4HBA   

**Figure 13:**
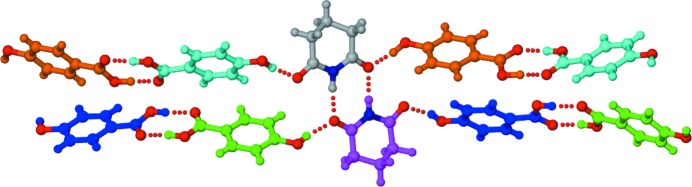
The structure of the 1:2 GM–4HBA co-crystal. Non-planar *T*_homo_-II units are formed by N—H⋯O dimers between symmetry-independent GM molecules (shown in different colours). Acid homodimers between independent 4HBA molecules are connected through O—H_hydroxy_⋯O=C_imide_ interactions. Dotted lines indicate hydrogen bonds.

Crystallization of a 1:1 GM–4HBA ground mixture from methanol resulted in a co-crystal with 1:2 stoichiometry. The structure displays non-planar *T*_homo_-II units formed by N—H⋯O dimers between GM molecules, and acid homodimers formed between 4HBA molecules connected through carbonyl–hydroxyl interactions (Fig. 13 ▶). The 4HBA molecules form two pairs of homodimers in which the component 4HBA molecules have different hydroxyl conformations (*cis* in one molecule and *trans* in the other, within a given pair).

#### 2.4.2. 1:1 GM–35DHBA co-crystal   

**Figure 14:**
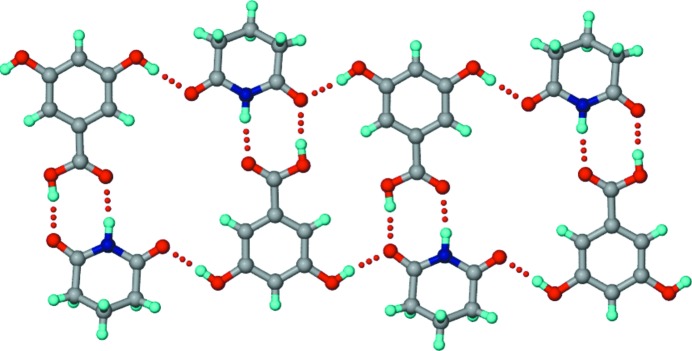
The structure of the 1:1 GM–35DHBA co-crystal. The glutarimide and 35DHBA molecules form *T*_hetero_ units through imide–acid ring dimers, which propagate through *meta* O—H_hydroxyl_⋯O=C_imide_ interactions. Dotted lines indicate hydrogen bonds.

Crystallization of a 1:1 GM–4HBA ground mixture from methanol resulted in a 1:1 co-crystal. The crystal structure includes *T*_hetero_ units consisting of imide–acid ring heterodimers which are propagated by hydrogen bonds between the peripheral carbonyls of GM and the *meta*-hydroxyl groups of 35DHBA (Fig. 14 ▶).

#### 2.4.3. GM–24DHBA/34DHBA/345THBA   

Moragues-Bartolome *et al.* (2012 ▶) reported the formation of a new solid for the GM–24DHBA combination but could not produce single crystals suitable for structure determination. Similarly, we could not obtain crystal structures of these combinations, but their distinct PXRD patterns compared with their parent compounds (see supporting information) establish them to be co-crystal-forming combinations.

### 2.5. Binary phase diagrams of eutectic-forming combinations   

**Figure 15:**
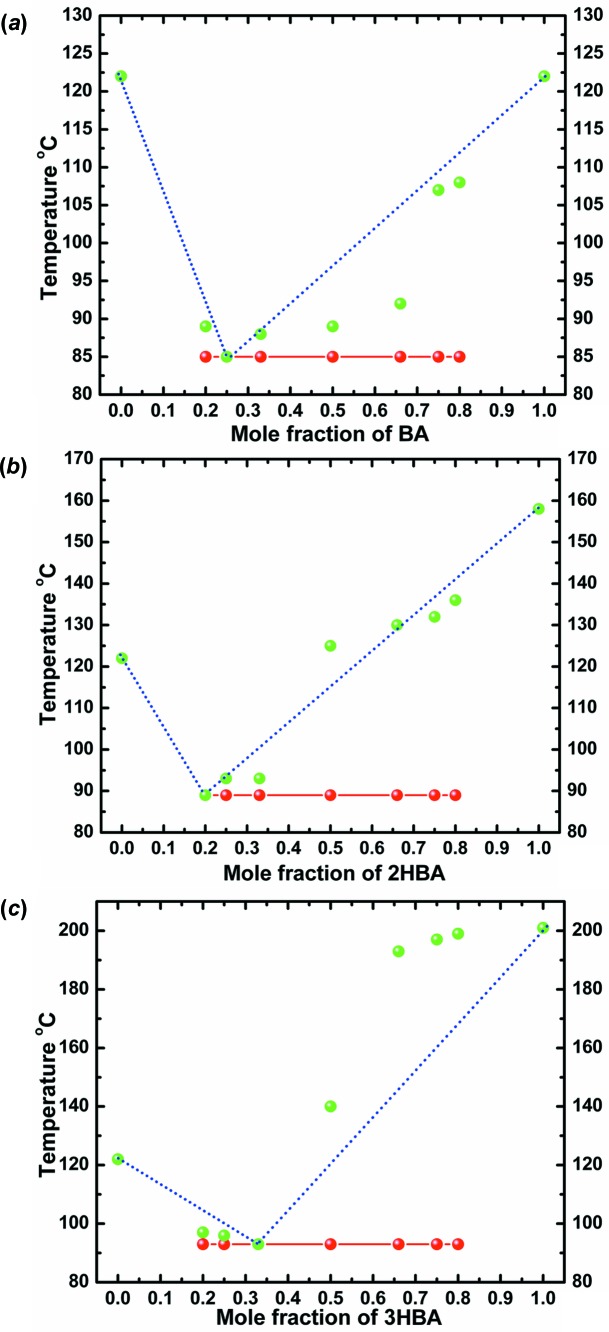
Binary phase diagrams of eutectic systems of (*a*) SM–BA, (*b*) SM–2HBA and (*c*) SM–3HBA combinations. Solidus points are shown in red and liquidus points in green.

Moragues-Bartolome *et al.* (2012 ▶) concluded that the benzoic acid–succinimide/glutarimide combination did not form co-crystals. We corroborate this result, but in addition can demonstrate the formation of eutectic mixtures by constructing phase diagrams. The thermal behaviour of different molar compositions (1:1, 1:2, 2:1, 1:3, 3:1, 1:4, 4:1) for each of the combinations was analysed on a melting-point apparatus and the solidus–liquidus events were plotted. Based on the single invariant low melting point observed in all compositions, and the characteristic ‘V’-type phase diagram, co-crystal formation in any stoichiometric ratio is ruled out. The eutectic composition for each of the combinations was determined from the meeting of the solidus and liquidus points. All three cyclic imides formed eutectics with benzoic acid and 2- and 3-hydroxybenzoic acids, and the phase diagrams are given in Figs. 15 ▶–17 ▶ ▶. The structural basis for the eutectic mixtures is the possibility of finite *T*_homo_ or *T*_hetero_ units, as discussed before.

## 3. Conclusions   

We have carried out an extensive study of the supramolecular compatibility of various cyclic imide–aromatic carboxylic acid combinations in terms of the formation of co-crystals and eutectic mixtures. Several co-crystals and eutectics were obtained, in accordance with our supramolecular design schematics. It appears convincing that, in general, all the cyclic imide–hydroxybenzoic acid co-crystals manifest as per Figs. 2 ▶ and 4 ▶. However, the co-crystal architecture schematized is an ideal situation and suits only 1:1 stoichiometries, if any. The strength and conformational flexibility associated with the hydroxyl group and the crystallization milieu factors (solvent, temperature, supersaturation *etc.*) facilitate co-crystal formation in different architectures (polymorphic arrangements), with different conformers (multiple stoichiometry), and sometimes including water of crystallization (Thakuria *et al.*, 2012 ▶). Earlier studies from our group have shown that the relative differences in the propensity to form supramolecular synthons and in the induction strength complementarity of hydrogen-bonding functional groups guide the formation of co-crystals and eutectics in a mutually exclusive manner for a given combination (Cherukuvada & Row, 2014 ▶; Prasad *et al.*, 2014 ▶). In this work, we have provided a rationale for their formation in the systems studied on the basis of an additional functional group (in this case hydroxyl) and its geometric disposition and resultant supramolecular effect in different combinations. The observation of a sharp melting point lower than those of the individual components, and the coexistence of individual components (as per PXRD patterns), in the medicinally relevant systems studied here strengthens the prospects of eutectics for pharmaceutical applications. This work improves our understanding of the requisites for selective co-crystal or eutectic formation for a combination with extensive hydrogen-bonding prospects.

## Supplementary Material

Crystal structure: contains datablock(s) global, SM-4HBA, SM-24DHBA, SM-34DHBA, SM-345THBAI, SM-345THBAII, SM-35DHBA, MM-4HBA, MM-24DHBA, MM-35DHBA, GM-4HBA, GM-35DHBA. DOI: 10.1107/S2052252515002651/bi5039sup1.cif

Structure factors: contains datablock(s) SM-4HBA. DOI: 10.1107/S2052252515002651/bi5039SM-4HBAsup2.hkl

Structure factors: contains datablock(s) SM-24DHBA. DOI: 10.1107/S2052252515002651/bi5039SM-24DHBAsup3.hkl

Structure factors: contains datablock(s) SM-34DHBA. DOI: 10.1107/S2052252515002651/bi5039SM-34DHBAsup4.hkl

Structure factors: contains datablock(s) SM-345THBAI. DOI: 10.1107/S2052252515002651/bi5039SM-345THBAIsup5.hkl

Structure factors: contains datablock(s) SM-345THBAII. DOI: 10.1107/S2052252515002651/bi5039SM-345THBAIIsup6.hkl

Structure factors: contains datablock(s) SM-35DHBA. DOI: 10.1107/S2052252515002651/bi5039SM-35DHBAsup7.hkl

Structure factors: contains datablock(s) MM-4HBA. DOI: 10.1107/S2052252515002651/bi5039MM-4HBAsup8.hkl

Structure factors: contains datablock(s) MM-24DHBA. DOI: 10.1107/S2052252515002651/bi5039MM-24DHBAsup9.hkl

Structure factors: contains datablock(s) mm-35DHBA. DOI: 10.1107/S2052252515002651/bi5039MM-35DHBAsup10.hkl

Structure factors: contains datablock(s) GM-4HBA. DOI: 10.1107/S2052252515002651/bi5039GM-4HBAsup11.hkl

Structure factors: contains datablock(s) GM-35DHBA. DOI: 10.1107/S2052252515002651/bi5039GM-35DHBAsup12.hkl

Supporting figures and tables. DOI: 10.1107/S2052252515002651/bi5039sup13.pdf

CCDC references: 1027541, 1027542, 1027543, 1027544, 1027545, 1027546, 1027547, 1027548, 1027549, 1027550, 1027551

## Figures and Tables

**Figure 16:**
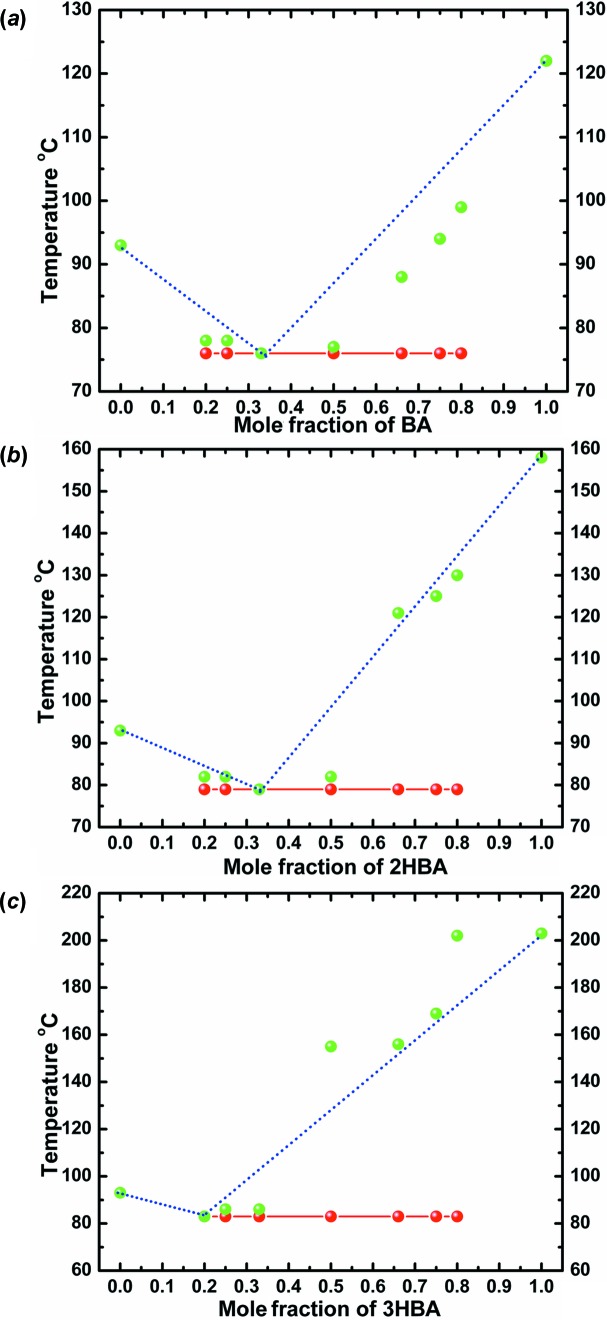
Binary phase diagrams of eutectic systems of (*a*) MM–BA, (*b*) MM–2HBA and (*c*) MM–3HBA combinations. Solidus points are shown in red and liquidus points in green.

**Figure 17:**
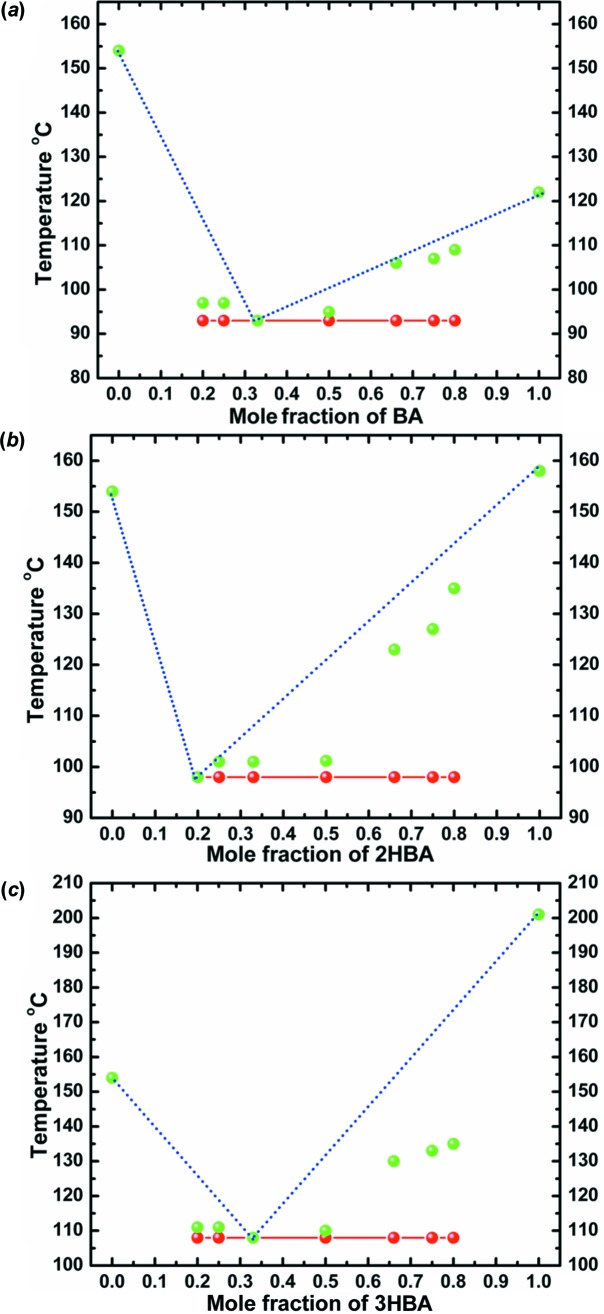
Binary phase diagrams of eutectic systems of (*a*) GM–BA, (*b*) GM–2HBA and (*c*) GM–3HBA combinations. Solidus points are shown in red and liquidus points in green.

**Table 1:** Crystallization results for the imidecarboxylic acid combinations

	Succinimide (SM)	Maleimide (MM)	Glutarimide (GM)
Benzoic acid (BA)	Eutectic	Eutectic	Eutectic
2-Hydroxybenzoic acid (2HBA)	Eutectic	Eutectic	Eutectic
3-Hydroxybenzoic acid (3HBA)	Eutectic	Eutectic	Eutectic
4-Hydroxybenzoic acid (4HBA)	1:1 Co-crystal	1:1 Co-crystal	1:2 Co-crystal
2,4-Dihydroxybenzoic acid (24DHBA)	1:1 Co-crystal (two polymorphs)	1:1 Co-crystal	Co-crystal (by PXRD)
3,4-Dihydroxybenzoic acid (34DHBA)	1:2 Co-crystal	Co-crystal (by PXRD)	Co-crystal (by PXRD)
3,5-Dihydroxybenzoic acid (35DHBA)	1:3:3 Co-crystal hydrate	1:3:3 Co-crystal hydrate	1:1 Co-crystal
3,4,5-Trihydroxybenzoic acid (345THBA)	2:1 Co-crystal (two polymorphs)	Co-crystal (by PXRD)	Co-crystal (by PXRD)
