# Supplementary material for: Do carboximide–carboxylic acid combinations form co-crystals? The role of hydroxyl substitution on the formation of co-crystals and eutectics
Source: IUCrJ. 2015 Apr 10;2(Pt 3):341–51. doi: 10.1107/S2052252515002651 (PMC4420544; doi:10.1107/S2052252515002651)
Supplement: Supplementary file 13 [file m-02-00341-sup13.pdf]

IUCrJ

Volume 2 (2015)

Supporting information for article:

Do Carboximide–Carboxylic Acid Combinations Form Co-crystals? Role of Hydroxyl Substitution on the Formation of Co-crystals and Eutectics

Ramanpreet Kaur, Raj Gautam, Suryanarayan Cherukuvada and Tayur N. Guru Row

2 Electronic Supplementary Information

Do Carboximide–Carboxylic acid combinations form Cocrystals? Role of Hydroxyl substitution on the formation of Cocrystals and Eutectics

Ramanpreet Kaur, Raj Gautam, Suryanarayan Cherukuvada, Tayur N. Guru Row*

Solid State and Structural Chemistry Unit, Indian Institute of Science, Bengaluru 560012, India
* Email: ssctng@sscu.iisc.ernet.in

Section S1: Experimental details – Pages S3-S5.

Section S2: Crystallographic tables of cocrystals, Tables S1-S3 – Pages S6-S8.
Table S1: Crystallographic parameters of succinimide cocrystals – Page S6.
Table S2: Crystallographic parameters of maleimide cocrystals – Page S7.
Table S3: Crystallographic parameters of glutarimide cocrystals – Page S8.

Section S3: PXRD pattern comparison of cocrystals with that of their respective parent materials, Figures S1-S5 – Pages S9-S13.
Figure S1: PXRD of maleimide–3,4-dihydroxybenzoic acid (**MM–34DHBA**) system – Page S9.
Figure S2: PXRD of maleimide–3,4,5-trihydroxybenzoic acid (**MM–345THBA**) system – Page S10.
Figure S3: PXRD of glutarimide–2,4-dihydroxybenzoic acid (**GM–24DHBA**) system – Page S11.
Figure S4: PXRD of glutarimide–3,4-dihydroxybenzoic acid (**GM–34DHBA**) system – Page S12.
Figure S5: PXRD of glutarimide–3,4,5-trihydroxybenzoic acid (**GM–345THBA**) system – Page S13.

Section S4: PXRD pattern comparison of eutectics with that of their respective parent materials, Figures S6-S8 – Pages S14-16.

Figure S6: PXRD of **(a)** succinimide–benzoic acid **(SM–BA)**, **(b)** succinimide–2-hydroxybenzoic acid **(SM–2HBA)** and **(c)** succinimide–3-hydroxybenzoic acid **(SM–3HBA)** systems – Page S14.

Figure S7: PXRD of **(a)** maleimide–benzoic acid **(MM–BA)**, **(b)** maleimide–2-hydroxybenzoic acid **(MM–2HBA)** and **(c)** maleimide–3-hydroxybenzoic acid **(MM–3HBA)** systems – Page S15.

Figure S8: PXRD of **(a)** glutarimide–benzoic acid **(GM–BA)**, **(b)** glutarimide–2-hydroxybenzoic acid **(GM–2HBA)** and **(c)** glutarimide–3-hydroxybenzoic acid **(GM–3HBA)** systems – Page S16.

References: Page S17.

Section S1: Experimental details

Materials: Commercially available compounds (Sigma-Aldrich, Bengaluru, India) were used without further purification. Solvents were of analytical or chromatographic grade and purchased from local suppliers.

Methods

Grinding: Compounds in molar ratios combined on the 100 mg scale were subjected to both neat and liquid-assisted grinding (with 1-2 mL acetonitrile) for 15 min using a mortar-pestle. The ground materials were analyzed by PXRD and melting point to ascertain the formation of the cocrystal or eutectic.

Evaporative Crystallization: Ground mixtures of different combinations were kept for crystallization at ambient conditions in different solvents viz. methanol, ethanol, propanol, acetone, Tetrahydrofuran (THF), 1,4-dioxane (14D), acetonitrile, ethyl acetate, DMF, DMSO etc. Majority of the cocrystal-forming combinations gave single crystals of cocrystals and eutectic-forming combinations separated into parent compounds upon crystallization.

1:1 SM–4HBA: Ground mixture of SM (10 mg, 0.1 mmol) and 4HBA (14 mg, 0.1 mmol) was dissolved in 5 mL methanol and left for slow evaporation at room temperature. Colorless block crystals were obtained after a few days upon solvent evaporation.

1:1 SM–24DHBA polymorphs: Ground mixture of SM (10 mg, 0.1 mmol) and 24DHBA (15.5 mg, 0.1 mmol) was dissolved in different solvents and left for slow evaporation at room temperature. Polymorph I crystallized as colorless needles from nitromethane and polymorph II as colorless block crystals from 14D after a few days upon solvent evaporation.

1:2 SM–34HBA: Ground mixture of SM (10 mg, 0.1 mmol) and 34DHBA (15.5 mg, 0.1 mmol) was dissolved in 5 mL acetonitrile and left for slow evaporation at room temperature. Colorless plate crystals were obtained after a few days upon solvent evaporation.

1:3:3 SM–35DHBA–H_2O: Ground mixture of SM (10 mg, 0.1 mmol) and 35DHBA (15.5 mg, 0.1 mmol) was dissolved in 5 mL methanol and left for slow evaporation at room temperature. Colorless block crystals were obtained after a few days upon solvent evaporation.

2:1 SM–345THBA polymorphs: Ground mixture of SM (10 mg, 0.1 mmol) and 345THBA (17 mg, 0.1 mmol) was dissolved in different solvents viz. methanol, ethanol, propanol, acetone, Tetrahydrofuran (THF), 1,4-dioxane (14D), acetonitrile, ethyl acetate, DMF, DMSO and left for slow evaporation at room temperature. Polymorph I crystallized as colorless needles from methanol, ethanol, acetone and THF and polymorph II as colorless needles from DMF and DMSO upon solvent evaporation.

1:1 MM–4HBA: Ground mixture of MM (10 mg, 0.1 mmol) and 4HBA (14 mg, 0.1 mmol) was dissolved in 5 mL THF and left for slow evaporation at room temperature. Colorless block crystals were obtained after a few days upon solvent evaporation.

1:1 MM–24DHBA: Ground mixture of MM (10 mg, 0.1 mmol) and 24DHBA (15.5 mg, 0.1 mmol) was dissolved in 5 mL 14D and left for slow evaporation at room temperature. Colorless plate crystals were obtained after a few days upon solvent evaporation.

1:3:3 MM–35DHBA–H_2O: Ground mixture of MM (10 mg, 0.1 mmol) and 35DHBA (15.5 mg, 0.1 mmol) was dissolved in 5 mL THF and left for slow evaporation at room temperature. Colorless plate crystals were obtained after a few days upon solvent evaporation.

1:2 GM–4HBA: Ground mixture of GM (11 mg, 0.1 mmol) and 4HBA (14 mg, 0.1 mmol) was dissolved in 5 mL n-propanol and left for slow evaporation at room temperature. Colorless needles crystals were obtained after a few days upon solvent evaporation.

1:1 GM–35DHBA: Ground mixture of GM (11 mg, 0.1 mmol) and 35DHBA (15.5 mg, 0.1 mmol) was dissolved in 5 mL methanol and left for slow evaporation at room temperature. Colorless needles crystals were obtained after a few days upon solvent evaporation.

Single crystal X-ray diffraction: X-ray reflections on suitable single crystals were collected on an Oxford Xcalibur (Mova) diffractometer equipped with an EOS CCD detector and a microfocus sealed tube using Mo Kα radiation (λ = 0.71073 Å). Data collection and reduction was performed using CrysAlisPro (version 1.171.36.32) [S1] and OLEX2 (version 1.2) [S2] was used to solve and refine the crystal structures. All non-hydrogen atoms were refined anisotropically. Hydrogen atoms on O and N were located from difference electron density maps (except in 1:3:3

SM–35DHBA–H_2O and 1:3:3 MM–35DHBA–H_2O where hydrogens were vaguely resolved) and all C–H atoms were fixed geometrically using HFIX command. The WinGX package [S3] was used for final refinement and production of CIFs and crystallographic tables.

Powder X-ray Diffraction: PXRD were recorded on PANalytical X'Pert diffractometer using Cu-Kα X-radiation (λ = 1.54056 Å) at 40 kV and 30 mA. X'Pert HighScore Plus (version 1.0d) [S4] was used to collect and plot the diffraction patterns. Diffraction patterns were collected over 2θ range of 5-40° using a step size of 0.06° 2θ and time per step of 1 sec.

Thermal analysis: Different compositions of eutectic-forming combinations were analyzed for their solidus-liquidus temperatures on a Labindia visual melting range apparatus (MR 13300710) equipped with a camera and a LCD monitor.

Packing Diagrams: X-Seed was used to prepare packing diagrams.[S5]

Section S2:

Table S1 Crystallographic parameters of succinimide cocrystals.

**Cocrystal**	**1:1 SM–4HBA**	**1:1 SM–24DHBA-II**	**1:2 SM–34DHBA**	**1:3:3 SM–35DHBA–H_2O**	**2:1 SM–345THBA-I**	**2:1 SM–345THBA-II**
Formula	$C_{11}H_{11}N_1O_5$	$C_{11}H_{11}N_1O_6$	$C_{18}H_{17}N_1O_{10}$	$C_{25}H_{23}N_1O_{17}$	$C_{15}H_{16}N_2O_9$	$C_{15}H_{16}N_2O_9$
Formula weight	237.21	253.21	407.33	609.44	368.30	368.30
**CCDC number**	**1027541**	**1027542**	**1027543**	**1027544**	**1027545**	**1027546**
Temperature (K)	100(2)	100(2)	100(2)	100(2)	130(2)	110(2)
R(int)	0.0341	0.0266	0.0395	0.0302	0.0496	0.0704
Crystal system	triclinic	triclinic	monoclinic	triclinic	Orthorhombic	triclinic
Space group	$P\bar{1}$	$P\bar{1}$	$P2_1/c$	$P\bar{1}$	$P2_12_12_1$	$P\bar{1}$
a (Å)	6.5133(3)	6.7358(8)	6.7323(2)	9.3161(5)	7.0213(3)	4.9225(4)
b (Å)	8.1853(5)	6.9119(8)	12.1142(5)	11.2092(3)	8.8214(4)	11.7839(10)
c (Å)	11.4965(6)	12.3937(9)	21.2077(8)	13.7362(7)	25.1416(2)	13.8540(16)
α (°)	103.458(5)	74.468(9)	90	102.926(3)	90	97.248(8)
β (°)	93.925(4)	85.298(8)	97.146(3)	104.398(4)	90	96.773(8)
γ (°)	113.018(5)	73.280(10)	90	96.571(3)	90	90.663(6)
Volume (Å^3)	539.85(6)	532.43(10)	1716.19(11)	1332.01(11)	1557.22(12)	791.35(13)
Z[S6]	4	4	12	14	12	6
Density (g cm^{-3})	1.46	1.58	1.58	1.52	1.57	1.55
μ (mm^{-1})	0.117	0.131	0.131	0.131	0.132	0.130
F (000)	248	264	848	632	768	384
$h_{min, max}$	-8,9	-8,8	-8,8	-10,11	-8,8	-6,6
$k_{min, max}$	-11,11	-8,6	-14,13	-8,13	-10,10	-14,14
$l_{min, max}$	-16,15	-14,13	-25,26	-16,14	-29,29	-17,17
No. of measured reflections	13046	4016	10777	9808	8817	11875
No. of unique reflections	1903	1861	3365	5225	2707	2784
No. of reflections used	1793	1690	2818	4753	2504	2267
No. of parameters	166	179	290	428	259	287
$R_{\_all}$, $R_{\_obs}$	0.032,0.031	0.058, 0.054	0.056, 0.044	0.081, 0.076	0.045,0.039	0.117,0.100
wR_{2_all}, wR_{2_obs}	0.084,0.082	0.147,0.145	0.096,0.091	0.191,0.189	0.096,0.091	0.237,0.231
$\Delta\rho_{min,max}$ (e Å^{-3})	-0.256,0.200	-0.370 ,0.545	-0.220,0.265	-0.328,0.738	-0.179,0.153	-0.429,0.451
GOOF	1.056	1.256	1.076	1.203	1.043	1.103

Table S2 Crystallographic parameters of maleimide cocrystals.

**Cocrystal**	**1:1 MM–4HBA**	**1:1 MM–24DHBA**	**1:3:3 MM–35DHBA–H_2O**
Formula	$C_{11}H_9N_1O_5$	$C_{11}H_9N_1O_6$	$C_{25}H_{19}N_1O_{17}$
Formula weight	235.19	251.19	605.41
**CCDC number**	**1027547**	**1027548**	**1027549**
Temperature (K)	100(2)	100(2)	100(2)
R(int)	0.0246	0.0409	0.082
Crystal system	monoclinic	monoclinic	triclinic
Space group	$P2_1/n$	*I2/a*	$P\bar{1}$
a (Å)	10.8426(8)	12.5506(4)	9.3796(10)
b (Å)	6.5202(4)	6.6807(2)	10.3981(12)
c (Å)	16.1326(13)	26.1586(8)	15.6415(16)
α (°)	90	90	80.620(9)
β (°)	106.391(8)	98.815(3)	72.913(9)
γ (°)	90	90	66.089(10)
Volume (Å^3)	1094.16(14)	2167.41(9)	1331.35(3)
Z	8	16	14
Density (gcm^{-3})	1.43	1.54	1.51
μ (mm^{-1})	0.115	0.128	0.131
F (000)	488	1040	624
$h_{min, max}$	-13,14	-15,15	-11,11
$k_{min, max}$	-8,8	-8,8	12,12
$l_{min, max}$	-22,21	-32,32	-19,18
No. of measured reflections	5081	21462	12476
No. of unique reflections	2375	2137	12476
No. of reflections used	1679	2002	4751
No. of parameters	166	179	396
R_{all}, R_{obs}	0.070, 0.048	0.057, 0.053	0.212, 0.110
wR_2_{all}, wR_2_{obs}	0.118,0.107	0.136, 0.133	0.321, 0.285
$\Delta\rho_{min,max}$ (e Å^{-3})	-0.170,0.152	-0.246,0.669	-0.490,0.854
GOOF	1.030	1.164	0.914

Table S3: Crystallographic parameters of glutarimide cocrystals.

**Cocrystal**	**1:2 GM–4HBA**	**1:1 GM–35DHBA**
Formula	$C_{19}H_{19}N_1O_8$	$C_{12}H_{13}N_1O_6$
Formula weight	389.35	267.23
**CCDC number**	**1027550**	**1027551**
Temperature (K)	100(2)	100(2)
R(int)	0.0585	0.0314
Crystal system	orthorhombic	triclinic
Space group	$Pca2_1$	$P\bar{1}$
a (Å)	40.6920(30)	6.6761(3)
b (Å)	5.4524(3)	9.1128 (4)
c (Å)	16.3546(9)	10.9447(4)
α (°)	90	93.397(3)
β (°)	90	107.694(3)
γ (°)	90	108.173(4)
Volume (Å^3)	3628.58(4)	593.92(13)
Z	24	4
Density (gcm^{-3})	1.43	1.49
μ (mm^{-1})	0.112	0.121
F (000)	1632	280
$h_{min, max}$	-40,50	-8,8
$k_{min, max}$	-6,6	-11,11
$l_{min, max}$	-15,20	-13,13
No. of measured reflections	15134	11839
No. of unique reflections	5638	2329
No. of reflections used	4868	2156
No. of parameters	594	212
$R_{\_all}$, $R_{\_obs}$	0.057, 0.048	0.033, 0.031
wR_{2_all}, wR_{2_obs}	0.108,0.102	0.088, 0.087
$\Delta\rho_{min,max}$ (e Å^{-3})	-0.218,0.220	-0.214,0.258
GOOF	1.027	1.058

Section S3: PXRD pattern comparison of cocrystals with that of their respective parent materials.

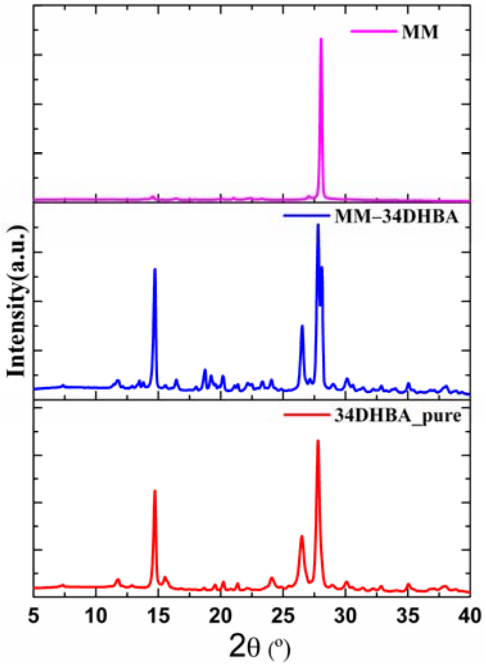

(a)

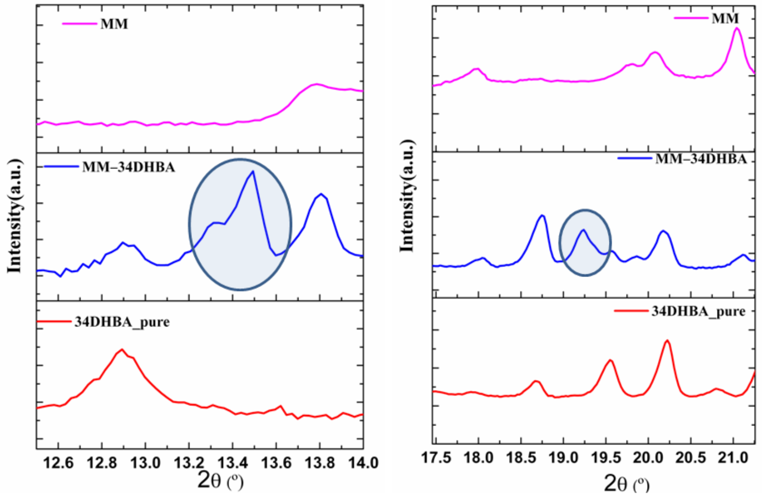

(b)

Figure S1 PXRD of 1:1 maleimide–3,4-dihydroxybenzoic acid ground material (blue) exhibits distinct diffraction peaks compared to the parent materials, maleimide (magenta) and 3,4-dihydroxybenzoic acid (red), and also their polymorphs and solvates found in the CSD.[S7] **(a)** 2 theta range in 5-40°. **(b)** New peaks about 13.4° and 19.2° for the combination indicate the formation of cocrystal.

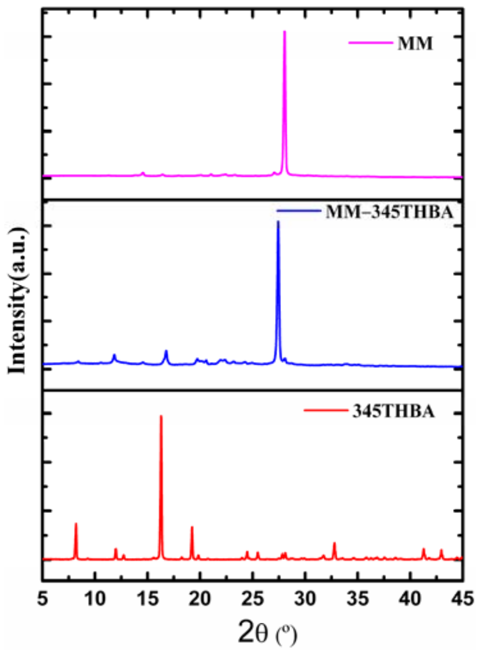

(a)

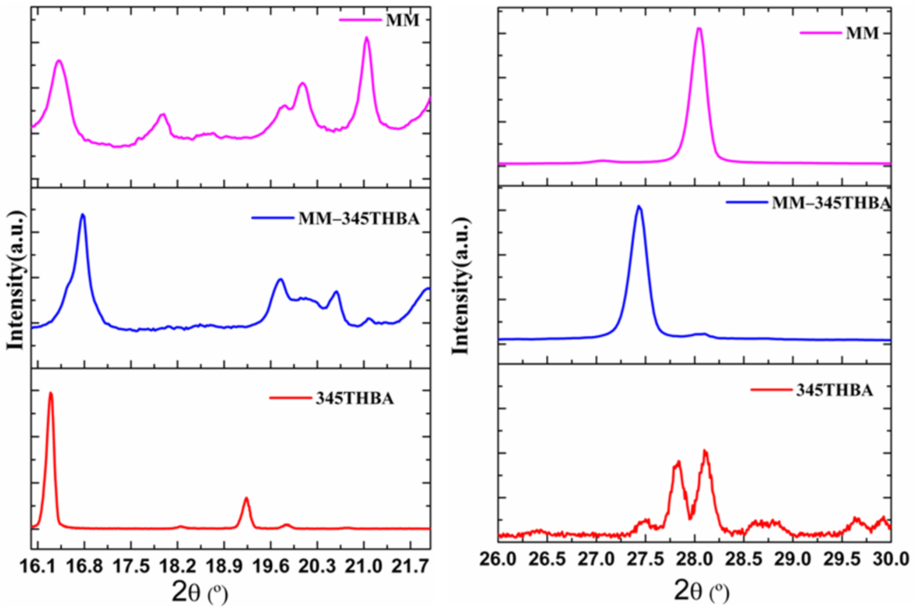

(b)

Figure S2 PXRD of 1:1 maleimide–3,4,5-trihydroxybenzoic acid ground material (blue) exhibits distinct diffraction peaks compared to the parent materials, maleimide (magenta) and 3,4,5-trihydroxybenzoic acid (red), and also their polymorphs and solvates found in the CSD. **(a)** 2 theta range in 5-40°. **(b)** New/distinct peaks about 16.8°, 20.6 and 27.5° for the combination indicate the formation of cocrystal.

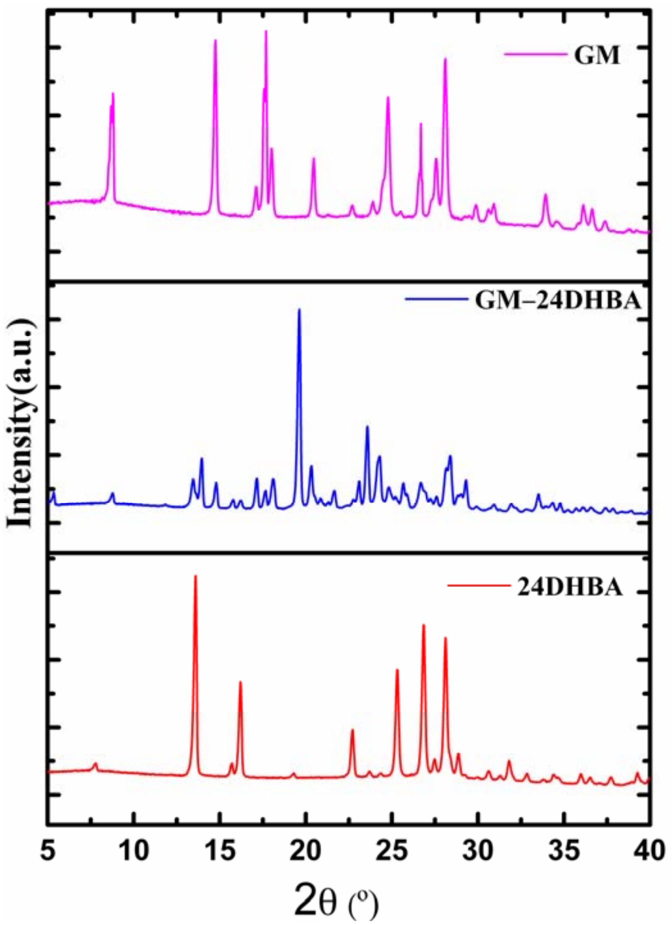

Figure S3 PXRD of 1:1 glutarimide–2,4-dihydroxybenzoic acid ground material (blue) exhibits distinct diffraction peaks compared to the parent materials, glutarimide (magenta) and 2,4-dihydroxybenzoic acid (red), and also their polymorphs and solvates found in the CSD.

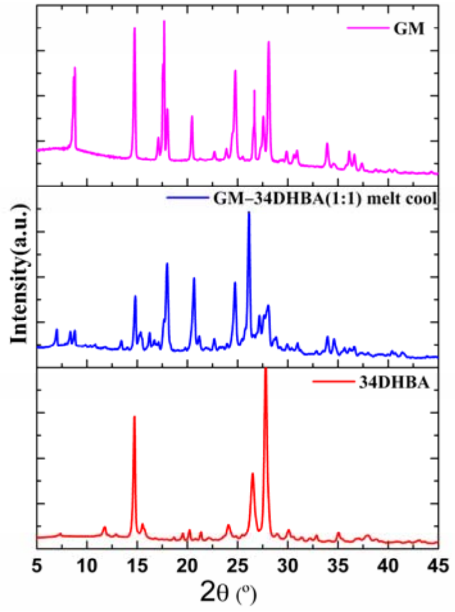

(a)

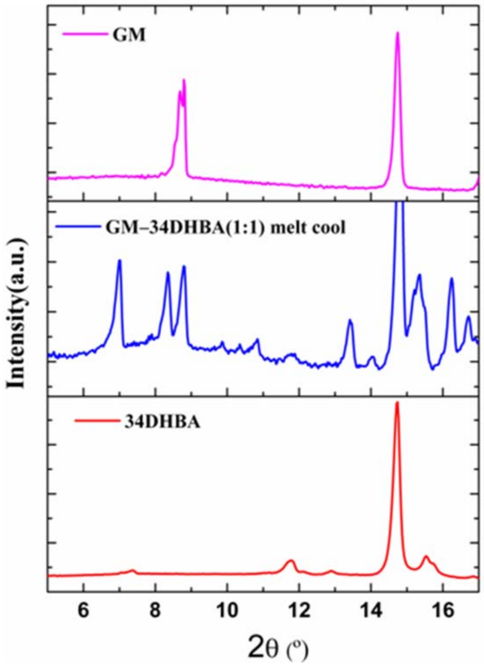

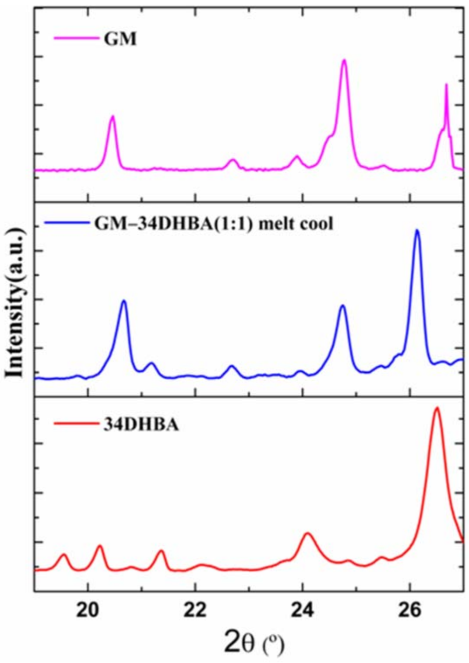

(b) **(c)**

Figure S4 PXRD of 1:1 glutarimide–3, 4-dihydroxybenzoic acid melt-crystallized material (blue) exhibits distinct diffraction peaks compared to the parent materials, glutarimide (magenta) and 3,4-dihydroxybenzoic acid (red), and also their polymorphs and solvates found in the CSD. (a) Shows full 2theta range, (b and c) shows enlarged PXRD in the range of 6-16° and 20-26°. The new peaks appear at 6.8°, 8.4°, 13.4°, 21.25°, 26.2°, 27.3°, 34.6° and 40.4°.

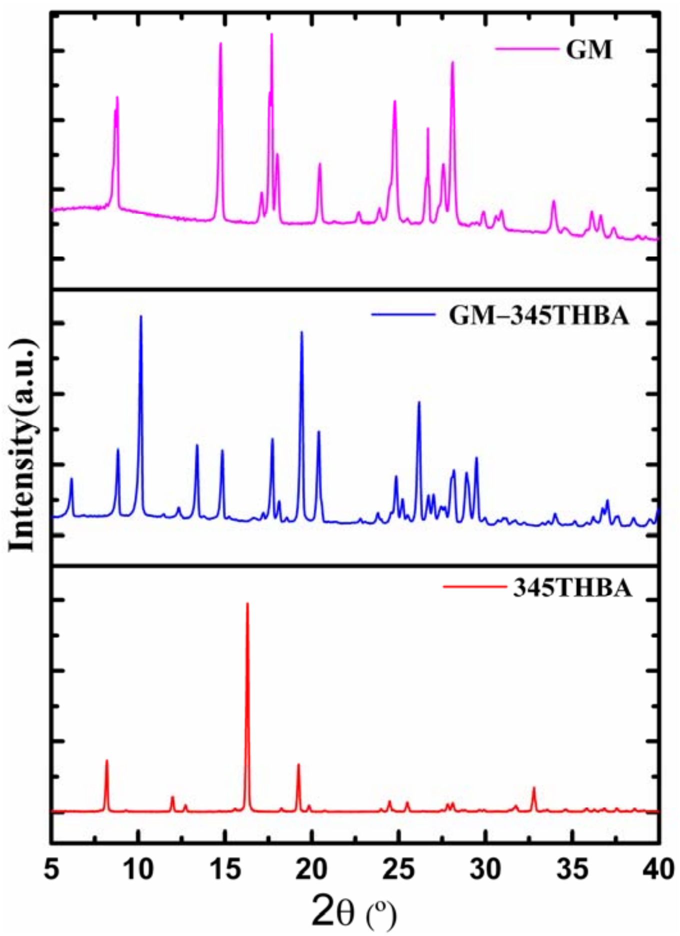

Figure S5 PXRD of 1:1 glutarimide–3,4,5-trihydroxybenzoic acid ground material (blue) exhibits distinct diffraction peaks compared to the parent materials, glutarimide (magenta) and 3,4,5-trihydroxybenzoic acid (red), and also their polymorphs and solvates found in the CSD.

Section S4: PXRD pattern comparison of eutectics with that of their respective parent materials.

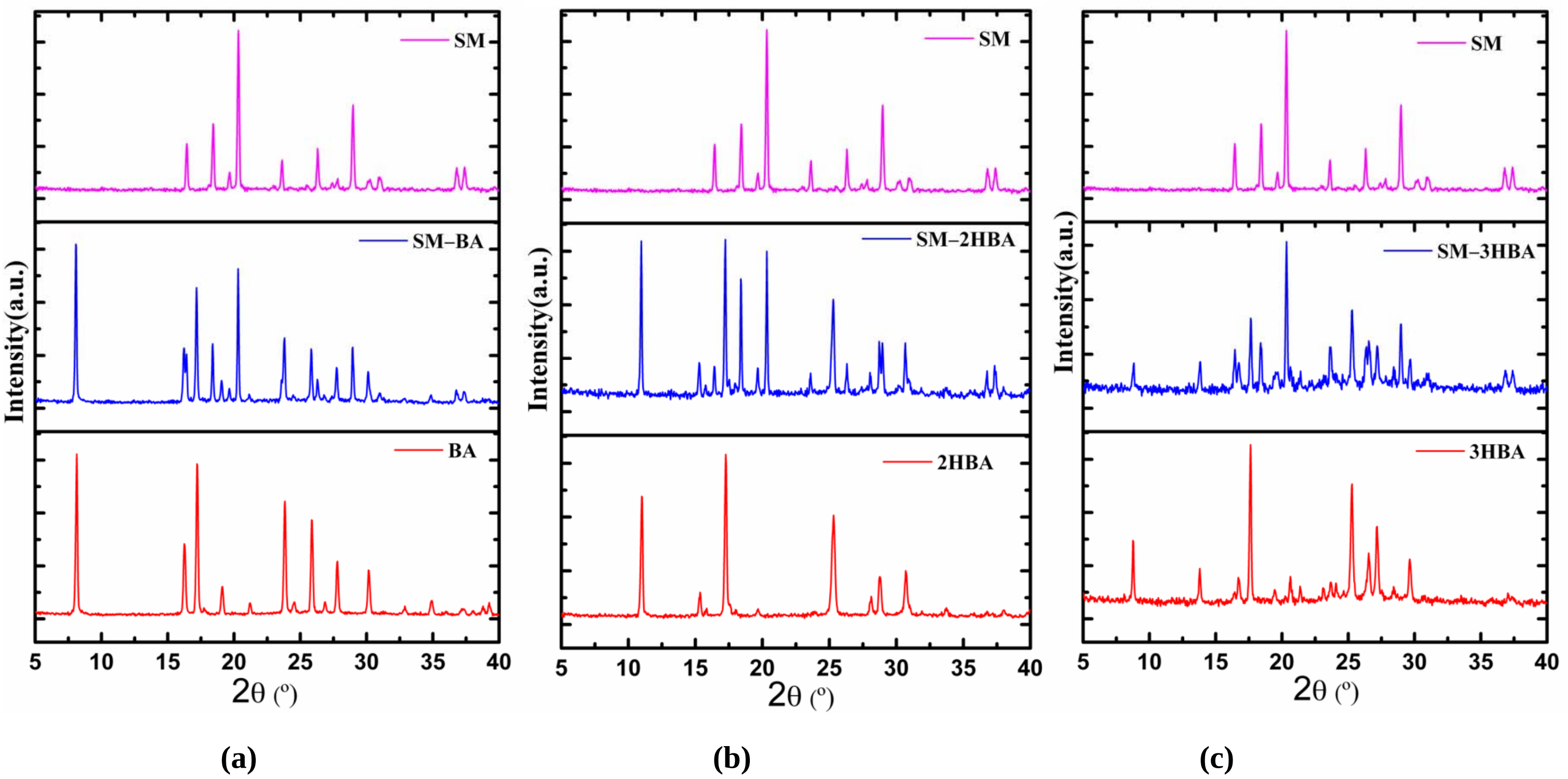

Figure S6 Comparison of PXRD patterns of **(a)** succinimide (magenta), benzoic acid (red) and their 1:1 ground material (blue) shows that the combination does not exhibit any new or distinct peaks characteristic of a cocrystal. Melting point analysis established the combination as a eutectic system. Similar observations can be seen for **(b)** succinimide–2-hydroxybenzoic acid and **(c)** succinimide–3-hydroxybenzoic acid systems.

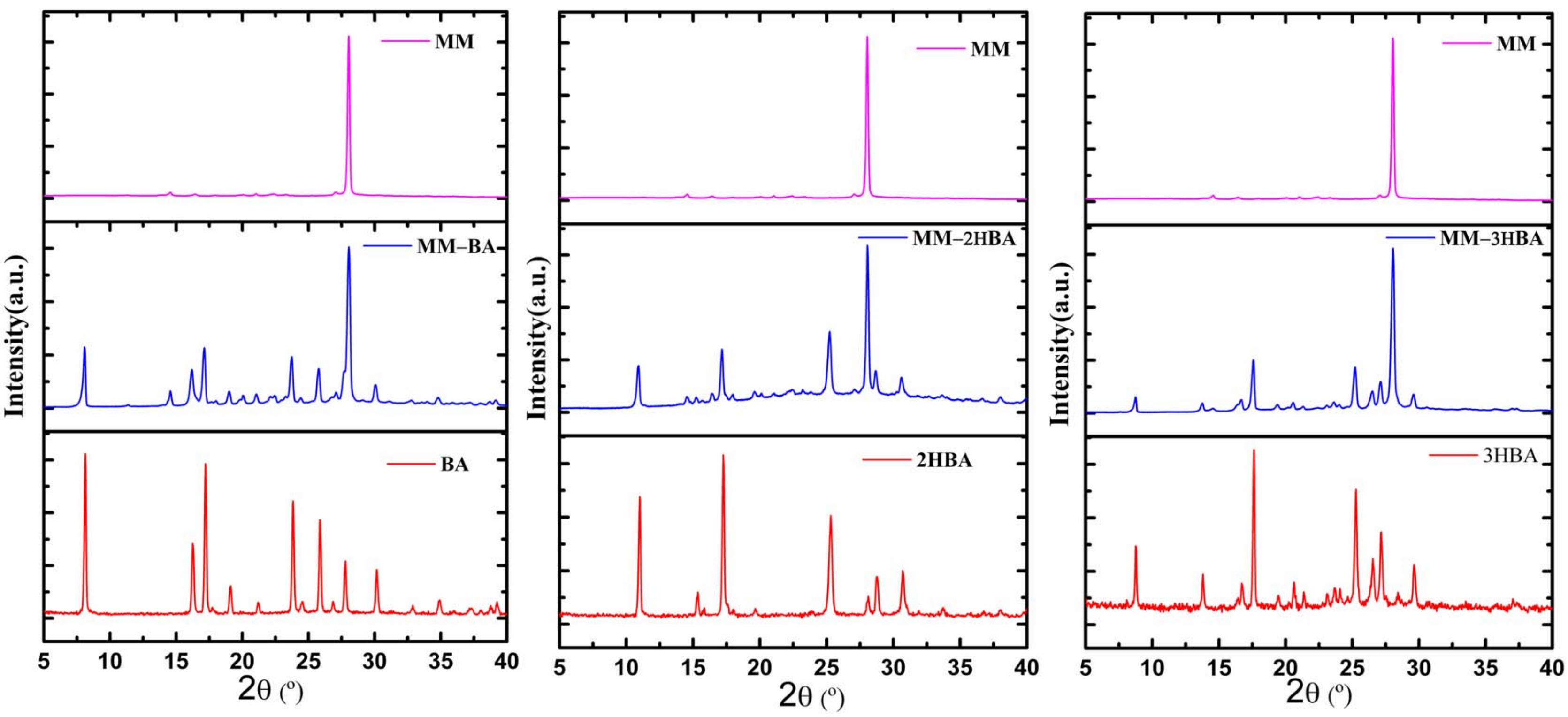

(a) (b) (c)

Figure S7 Comparison of PXRD patterns of **(a)** maleimide (magenta), benzoic acid (red) and their 1:1 ground material (blue) shows that the combination does not exhibit any new or distinct peaks characteristic of a cocrystal. Melting point analysis established the combination as a eutectic system. Similar observations can be seen for **(b)** maleimide–2-hydroxybenzoic acid and **(c)** maleimide–3-hydroxybenzoic acid systems.

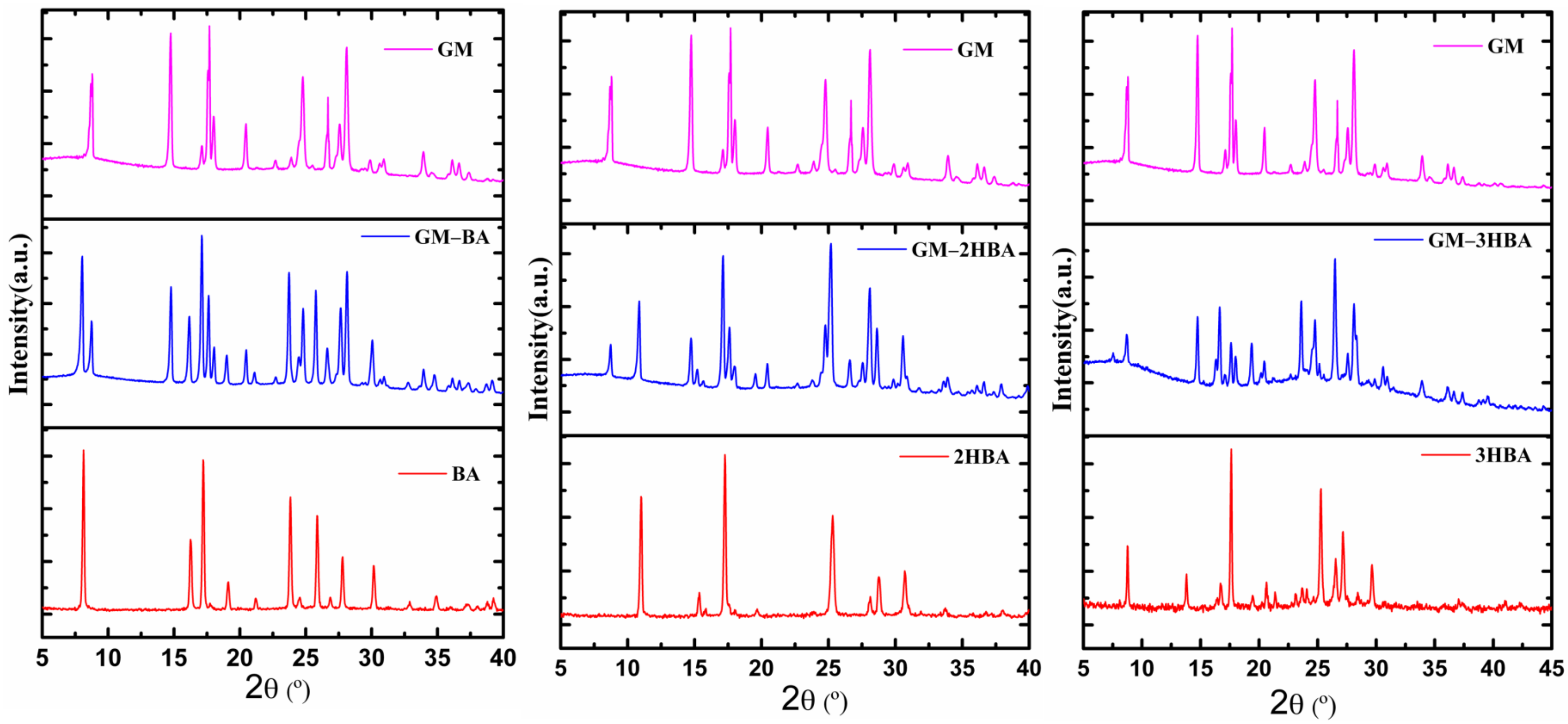

(a) **(b)** **(c)**

Figure S8 Comparison of PXRD patterns of **(a)** glutarimide (magenta), benzoic acid (red) and their 1:1 ground material (blue) shows that the combination does not exhibit any new or distinct peaks characteristic of a cocrystal. Melting point analysis established the combination as a eutectic system. Similar observations can be seen for **(b)** glutarimide–2-hydroxybenzoic acid and **(c)** glutarimide–3-hydroxybenzoic acid systems.

References:

S1. CrysAlisPro, ver. 1.171.36.32, (2011).Agilent Technologies UK Ltd: Yarnton, England.

S2. Dolomanov, O. V., Blake, A. J., Champness, N. R. & Schröder, M. (2003). *J. Appl. Cryst.* **36**, 1283–1284.

S3. Farrugia, L. J. (1999). *J. Appl. Cryst.* **32**, 837–838.

S4. X'Pert HighScore Plus, *The complete powder analysis tool*, PANalytical B. V. 2003.

S5. Barbour, L. J. (1999). *X-Seed, Graphical Interface to SHELX-97 and POV-Ray*, *Program for Better Quality of Crystallographic Figures*; University of Missouri-Columbia, Columbus, MO.

S6. Van Eijck, B. P. & Kroon, J. (2000). *Acta Cryst.B* **56**, 535–542.

$Z = Z''$ (no. of crystallographically non-equivalent molecules of any type in the asymmetric unit) × no. of independent general positions of the space group.

S7. Cambridge Structural Database, ver. 5.35, ConQuest 1.16, www.ccdc.cam.ac.uk.
